# Biochemical and molecular characterization of *Campylobacter fetus* isolates from bulls subjected to bovine genital campylobacteriosis diagnosis in Spain

**DOI:** 10.1186/s12917-024-03970-8

**Published:** 2024-04-02

**Authors:** Nerea Pena-Fernández, Nekane Kortabarria, Ana Hurtado, Medelin Ocejo, Marcelo Fort, Iratxe Pérez-Cobo, Esther Collantes-Fernández, Gorka Aduriz

**Affiliations:** 1 SERIDA, Servicio Regional de Investigación y Desarrollo Agroalimentario, Carretera de Oviedo, S/N, Villaviciosa, 33300 Spain; 2 Department of Animal Health, NEIKER-Basque Institute for Agricultural Research and Development, Basque Research and Technology Alliance (BRTA), Parque Científico y Tecnológico de Bizkaia, P812, Derio, 48160 Spain; 3 SALUVET, Animal Health Department, Faculty of Veterinary Sciences, Complutense University of Madrid, Ciudad Universitaria S/N, Madrid, 28040 Spain; 4 Central Veterinary-Animal Health Laboratory (LCV), Ctra. Madrid-Algete Km. 8.00, Algete, 28110 Spain; 5 Faculty of Veterinary Sciences, SALUVET-Innova S.L, Complutense University of Madrid, Ciudad Universitaria S/N, Madrid, 28040 Spain

**Keywords:** *Campylobacter fetus venerealis*, Biochemical tests, PCR, Whole Genome Sequencing (WGS), Bovine Genital Campylobacteriosis (BGC)

## Abstract

**Background:**

Bovine genital campylobacteriosis (BGC) is caused by *Campylobacter fetus* subsp. *venerealis* (*Cfv*) including its biovar *intermedius* (*Cfvi*). This sexually transmitted disease induces early reproductive failure causing considerable economic losses in the cattle industry. Using a collection of well-characterized isolates (*n* = 13), *C. fetus* field isolates (*n* = 64) and saprophytic isolates resembling *Campylobacter* (*n* = 75) obtained from smegma samples of breeding bulls, this study evaluated the concordance of the most used phenotypic (H_2_S production in cysteine medium and 1% glycine tolerance) and molecular (PCR) methods for the diagnosis of BGC and assessed possible cross-reactions in the molecular diagnostic methods.

**Results:**

Characterization at the subspecies level (*fetus* vs. *venerealis)* of *C. fetus* isolated from bull preputial samples using phenotypic and molecular (PCR targeting *nahE* and *ISCfe1*) methods showed moderate concordance (κ = 0.462; CI: 0.256–0.669). No cross-reactions were observed with other saprophytic microaerophilic species or with other *Campylobacter* species that can be present in preputial samples. Whole genome sequencing (WGS) of discrepant isolates showed 100% agreement with PCR identification. For the differentiation of *Cfv* biovars, comparison of the H_2_S test (at 72 h and 5 days of incubation) and a PCR targeting the L-cysteine transporter genes showed higher concordance when H_2_S production was assessed after 5 days (72 h; κ = 0.553, 0.329–0.778 CI *vs*. 5 days; κ = 0.881, 0.631–1 CI), evidencing the efficacy of a longer incubation time.

**Conclusions:**

This study confirmed the limitations of biochemical tests to correctly identify *C. fetus* subspecies and biovars. However, in the case of biovars, when extended incubation times for the H_2_S test (5 days) were used, phenotypic identification results were significantly improved, although PCR-based methods produced more accurate results. Perfect agreement of WGS with the PCR results and absence of cross-reactions with non-*C. fetus* saprophytic bacteria from the smegma demonstrated the usefulness of these methods. Nevertheless, the identification of new *C. fetus* subspecies-specific genes would help to improve BGC diagnosis.

**Supplementary Information:**

The online version contains supplementary material available at 10.1186/s12917-024-03970-8.

## Background

*Campylobacter fetus* subspecies *venerealis* (*Cfv*) and its biovar *intermedius* (*Cfvi*) [1] are the causative agents of Bovine Genital Campylobacteriosis (BGC), a sexually transmitted disease (STD) of cattle listed by the World Organisation for Animal Health (WOAH) [2–5] and included in the European legislation (Regulation (EU) 2016/429) that governs the trade of frozen bovine semen. The main route of *Cfv* transmission is during coitus, although transmission can also occur during artificial insemination via contaminated material or semen [4]. Bulls do not usually show clinical signs and can become chronic carriers. Heifers and cows often develop a self-limiting genital infection that causes endometritis and salpingitis, and can result in infertility, early reproductive failure and abortions [4, 6, 7]. Herds with BGC often have reduced reproductive efficiency due to lower pregnancy rates, higher number of services per conception and longer calving intervals, resulting in large economic losses in the beef cattle sector [3, 8, 9]. BGC is widely distributed in areas where cattle are extensively managed and natural breeding is practised [8, 9]. In some areas of Spain, BGC remains endemic with individual and herd prevalence of 7.7% and 12.2%, respectively [10]. However, the lack of monitoring programmes for this disease in many countries makes it difficult to estimate the prevalence rates of BGC world-wide.

The WOAH recommends microbiological culture followed by subsequent phenotypic characterization of *C. fetus* to ensure good health status before the movement of cattle [2]. However, *Cfv* is a fastidious growing microorganism with particular physiological characteristics and special nutritional requirements. These characteristics and the common presence of other competing saprophytic bacteria in preputial samples compromise the sensitivity and specificity of *Cfv* detection by bacterial culture [7, 11]. Moreover, another subspecies of *C. fetus*, *C. fetus* subsp. *fetus* (*Cff*) colonizes the intestinal tract of ruminants and occasionally the preputial cavity, causing sporadic abortion in cattle [7, 12]. The correct identification of *Cfv* and *Cff* is crucial for the implementation of effective BGC control and eradication programmes. *C. fetus* subspecies differentiation is possible by biochemical tests, with the 1% glycine tolerance test and hydrogen sulphide (H_2_S) production being the most relevant (*Cfv* is negative while *Cff* shows positive results to both tests) [1, 2]. However, since some *Cfv* strains can acquire glycine tolerance through phage-mediated transduction processes and mutations [13], these biochemical test may lead to misidentification. Furthermore, *Cfvi* strains exhibit biochemical characteristics intermediate between *Cfv* and *Cff*, which include low glycine tolerance and H_2_S production, that could lead to misidentification in diagnosis [1, 4].

Conventional culture-based methods are time-consuming and laborious, and several PCR methods have been developed for the BGC diagnosis. The most reliable PCR methods for *C. fetus* subspecies differentiation are those targeting the *nahE* gene specific of *C. fetus* and the *Cfv-*specific insertion element *ISCfe1* [14, 15]. However, none of the methods so far described demonstrated complete accuracy in the identification of *C. fetus* strains at the subspecies level [7, 14] and cross-reactions were also found with *Campylobacter hyointestinalis* isolates that carry the *ISCfe1* insertion element [14]. In addition, molecular differentiation between *Cfv* biovars is achieved by PCR targeting the L-cysteine (L-Cyst) ABC transporter genes, which are directly related to the H_2_S production typical of *Cfvi* isolates [16].

Comparative analyses of biochemical and molecular identification methodologies for *C. fetus* subspecies and *Cfv* biovars differentiation, using a wide range of field isolates, are scarce. Therefore, the aim of this study was to compare phenotypic methods and PCR assays based on the *nahE* and *ISCfe1* [14, 15] and the L-Cyst ABC transporter gene [16] targets to differentiate *Cff, Cfv* and *Cfvi*, using a wide panel of *C. fetus* control and field isolates. In addition, we studied the impact of longer incubation time on H_2_S production on *Cfvi* identification. The isolates with discordant results were whole genome sequenced (WGS) for a more accurate identification. The performance of the PCR assays used for the identification of *C. fetus* subspecies was also evaluated using saprophytic bacteria with similar morphology and motility to *Campylobacter* isolated from the preputial cavity of breeding bulls to further examine possible occurrence of cross-amplification reactions.

## Results

### Phenotypic characterization of *C. fetus* isolates by biochemical tests

The expected results of biochemical tests for *Cff*, *Cfv* and related species as described by WOAH are detailed in Table S1. Based on the results of the 1% glycine tolerance and H_2_S production tests, 12 of the 13 *C. fetus* control strains tested (92.3%) were identified as *Cff* and 1 (7.7%) as *Cfv*. Notably, although the final identification of the control strains was the same regardless of the incubation time for the H_2_S test (72 h and 5 days), there were 3 *Cff* strains (*Cff*1, *Cff*2 and *Cff*-C0075) that at 72 h of incubation did not produce H_2_S, while at 5 days one of the strains was H_2_S-producer (*Cff*1). The only misidentified control isolate was *Cfvi3*, which showed tolerance to 1% glycine and H_2_S production after 5 days therefore being biochemically identified as *Cff* (Table 1). Finally, the *C. hyointestinalis* strain (*C. hyo hyo*) was classified as non-*C. fetus* (Table 1). Regarding the 64 *C. fetus* field strains, the analysis of 1% glycine tolerance and H_2_S production after 72 h of incubation, identified the 14.1% (9/64) of strains as *Cff* (one of them as non H_2_S-producer: Sal2), 29.7% (19/64) as *Cfv* and 56.2% (36/64) as *Cfvi*. However, 8 strains considered as *Cfv* at 72 h of incubation (H_2_S non-producers) were reclassified as *Cfvi* after 5 days of incubation (H_2_S producers), resulting in 17.2% (11/64) *Cfv* and 68.7% (44/64) *Cfvi* strains (Table 1).

**Table 1:** Phenotypic and molecular identification of *C. fetus* control and field isolates

	**Sample**	**Phenotypic identification tests**				**Molecular (PCR) identification tests**		
		**H**_**2**_**S 72 h**^**a**^	**H**_**2**_**S 5 days**^**b**^	**Glycine 1%**	**Phenotype**^**c**^	**PCR-1**^**d**^	**PCR-2**^**e**^	**PCR-3**^**f**^
**Control strains**	*Cfv ATCC 25922*	-	-	-	*Cfv*	*Cfv*	*Cfv*	*Cfv*
	***Cfvi3***	-	+	+	***Cff***	***Cfv***	***Cfv***	*Cfvi*
	*Cff1*	-	+	+	*Cff*	*Cff*	*Cff*	nd
	*Cff2*	-	-	+	*Cff*	*Cff*	*Cff*	nd
	*Cff C0075*	-	-	+	*Cff*	*Cff*	*Cff*	nd
	*Cff C0011*	+	+	+	*Cff*	*Cff*	*Cff*	nd
	*Cff C0023*	+	+	+	*Cff*	*Cff*	*Cff*	nd
	*Cff C0024*	+	+	+	*Cff*	*Cff*	*Cff*	nd
	*Cff C0037*	+	+	+	*Cff*	*Cff*	*Cff*	nd
	*Cff C0048*	+	+	+	*Cff*	*Cff*	*Cff*	nd
	*Cff C0054*	+	+	+	*Cff*	*Cff*	*Cff*	nd
	*Cff C0173*	+	+	+	*Cff*	*Cff*	*Cff*	nd
	*Cff C0228*	+	+	+	*Cff*	*Cff*	*Cff*	nd
	*C. hyo hyo*	nd	nd	+	No* C. fetus*	No* C. fetus*	No* C. fetus*	nd
***C. fetus* field isolates**	SAL 2	-	-	+	*Cff*	*Cff*	*Cff*	nd
	C1. 54	+	+	+	*Cff*	*Cff*	*Cff*	nd
	C2.39	+	+	+	*Cff*	*Cff*	*Cff*	nd
	**C2.6**	+	+	+	***Cff***	***Cfv***	***Cfv***	*Cfvi*
	**C3.10**	+	+	+	***Cff***	***Cfv***	***Cfv***	*Cfvi*
	**C3.4**	+	+	+	***Cff***	***Cfv***	***Cfv***	*Cfvi*
	**C3.46**	+	+	+	***Cff***	***Cfv***	***Cfv***	*Cfvi*
	**C3.52**	+	+	+	***Cff***	***Cfv***	***Cfv***	*Cfvi*
	**SAL 3**	+	+	+	***Cff***	***Cfv***	***Cfv***	*Cfvi*
	C1. 30	-	-	-	*Cfv*	*Cfv*	*Cfv*	*Cfv*
	C1. 49	-	-	-	*Cfv*	*Cfv*	*Cfv*	*Cfv*
	C1. 55	-	-	-	*Cfv*	*Cfv*	*Cfv*	*Cfv*
	C1. 70	-	-	-	*Cfv*	*Cfv*	*Cfv*	*Cfv*
	C2.30	-	-	-	*Cfv*	*Cfv*	*Cfv*	*Cfv*
	C3.49	-	-	-	*Cfv*	*Cfv*	*Cfv*	*Cfv*
	**C1. 43**	-	-	-	***Cfv***	*Cfv*	*Cfv*	***Cfvi***
	C1. 59	-	-	-	*Cfv*	*Cfv*	*Cfv*	*Cfv*
	C1. 84	-	-	-	*Cfv*	*Cfv*	*Cfv*	*Cfv*
	**C3.5**	-	-	-	***Cfv***	*Cfv*	*Cfv*	***Cfvi***
	SAL 5	-	-	-	*Cfv*	*Cfv*	*Cfv*	*Cfv*
	C1. 41	-	+	-	*Cfvi*	*Cfv*	*Cfv*	*Cfvi*
	C1. 56	-	+	-	*Cfvi*	*Cfv*	*Cfv*	*Cfvi*
	C2.1	-	+	-	*Cfvi*	*Cfv*	*Cfv*	*Cfvi*
	C2.31	-	+	-	*Cfvi*	*Cfv*	*Cfv*	*Cfvi*
	C3.63	-	+	-	*Cfvi*	*Cfv*	*Cfv*	*Cfvi*
	C1. 32	-	+	-	*Cfvi*	*Cfv*	*Cfv*	*Cfvi*
	C1. 67	-	+	-	*Cfvi*	*Cfv*	*Cfv*	*Cfvi*
	C1. 75	-	+	-	*Cfvi*	*Cfv*	*Cfv*	*Cfvi*
	C1. 24	+	+	-	*Cfvi*	*Cfv*	*Cfv*	*Cfvi*
	C1. 65	+	+	-	*Cfvi*	*Cfv*	*Cfv*	*Cfvi*
	C1. 69	+	+	-	*Cfvi*	*Cfv*	*Cfv*	*Cfvi*
	C1. 73	+	+	-	*Cfvi*	*Cfv*	*Cfv*	*Cfvi*
	C1. 85	+	+	-	*Cfvi*	*Cfv*	*Cfv*	*Cfvi*
	C1.91	+	+	-	*Cfvi*	*Cfv*	*Cfv*	*Cfvi*
	C1.96	+	+	-	*Cfvi*	*Cfv*	*Cfv*	*Cfvi*
	C2.12	+	+	-	*Cfvi*	*Cfv*	*Cfv*	*Cfvi*
	C2.17	+	+	-	*Cfvi*	*Cfv*	*Cfv*	*Cfvi*
	C2.21	+	+	-	*Cfvi*	*Cfv*	*Cfv*	*Cfvi*
	C2.32	+	+	-	*Cfvi*	*Cfv*	*Cfv*	*Cfvi*
	C2.7	+	+	-	*Cfvi*	*Cfv*	*Cfv*	*Cfvi*
	C2.8	+	+	-	*Cfvi*	*Cfv*	*Cfv*	*Cfvi*
	C3.6	+	+	-	*Cfvi*	*Cfv*	*Cfv*	*Cfvi*
	C1. 40	+	+	-	*Cfvi*	*Cfv*	*Cfv*	*Cfvi*
	C1. 58	+	+	-	*Cfvi*	*Cfv*	*Cfv*	*Cfvi*
	C1. 60	+	+	-	*Cfvi*	*Cfv*	*Cfv*	*Cfvi*
	C1. 76	+	+	-	*Cfvi*	*Cfv*	*Cfv*	*Cfvi*
	C1. 87	+	+	-	*Cfvi*	*Cfv*	*Cfv*	*Cfvi*
	C1. 88	+	+	-	*Cfvi*	*Cfv*	*Cfv*	*Cfvi*
	C1.92	+	+	-	*Cfvi*	*Cfv*	*Cfv*	*Cfvi*
	C2.14	+	+	-	*Cfvi*	*Cfv*	*Cfv*	*Cfvi*
	C2.15	+	+	-	*Cfvi*	*Cfv*	*Cfv*	*Cfvi*
	C2.2	+	+	-	*Cfvi*	*Cfv*	*Cfv*	*Cfvi*
	C2.22	+	+	-	*Cfvi*	*Cfv*	*Cfv*	*Cfvi*
	C2.3	+	+	-	*Cfvi*	*Cfv*	*Cfv*	*Cfvi*
	C2.33	+	+	-	*Cfvi*	*Cfv*	*Cfv*	*Cfvi*
	C2.35	+	+	-	*Cfvi*	*Cfv*	*Cfv*	*Cfvi*
	C2.36	+	+	-	*Cfvi*	*Cfv*	*Cfv*	*Cfvi*
	C2.37	+	+	-	*Cfvi*	*Cfv*	*Cfv*	*Cfvi*
	C3.15	+	+	-	*Cfvi*	*Cfv*	*Cfv*	*Cfvi*
	C3.17	+	+	-	*Cfvi*	*Cfv*	*Cfv*	*Cfvi*
	C3.44	+	+	-	*Cfvi*	*Cfv*	*Cfv*	*Cfvi*
	C3.62	+	+	-	*Cfvi*	*Cfv*	*Cfv*	*Cfvi*
	SAL 1	+	+	-	*Cfvi*	*Cfv*	*Cfv*	*Cfvi*
	SAL 4	+	+	-	*Cfvi*	*Cfv*	*Cfv*	*Cfvi*

Strains with discordant phenotypic and molecular identification results have been marked in bold

*nd* not determined,* Cff Camylobacter fetus* subsp. *fetus*, *Cfv Camylobacter fetus* subsp. *venerealis*, *Cfvi Camylobacter fetus* subsp. *venerealis* bv*. intermedius, C. hyo hyo Campylobacter hyointestinalis* subsp. *hyointestinalis*

^a, b^H_2_S production by lead acetate strips at 72 h and 5 days

^c^Phenotype results are based on the glycine tolerance test and H_2_S production after 5 days

^d^PCR-1: PCR described by Abril et al., for *Cfv* and *Cff* differentiation [15]

^e^PCR-2: Real time PCR described by van der Graaf et al., for *Cfv* and *Cff* differentiation [14]

^f^PCR-3: PCR described by Farace et al., for *Cfv* and *Cfvi* differentiation [16]

Regarding other phenotypic tests (growth at 42 °C, oxidase activity, catalase production and tolerance to 3.5% NaCl), the control strains *Cfv* ATCC 25922 and *Cfvi3* showed no growth at 42 °C and in medium with 3.5% NaCl and were positive for oxidase and catalase activity. In contrast, all *Cff* control strains grew at 42 ºC and showed identical results to *Cfv* ATCC 25922 and *Cfvi3* in all other tests (Table S2). Finally, the *C. hyointestinalis* strain tested positive to growth at 42 °C and oxidase activity and showed intolerance to 3.5% NaCl. However, it did not produce catalase, which is discordant with the expected result according to WOAH (Tables S1 and S2). Concerning the field isolates, all of them were oxidase and catalase producers and did not tolerate 3.5% NaCl. However, variable results were observed regarding their ability to grow at 42 °C. Thus, 45.5% (5/11) and 56.8% (25/44) of the field isolates biochemically characterized as *Cfv* and *Cfvi*, respectively, were able to grow at 42 °C, a typical characteristic of intestinal *Campylobacter* species. Finally, 100% of the *Cff* field isolates showed the same results as the control *Cff* isolates based on these tests (Table S2).

### Identification of *C. fetus* subspecies and biovar by PCR assays

Identification of *C. fetus* control strains at the subspecies level produced the same results (2 *Cfv* and 11 *Cff*) by either using PCR-1 or PCR-2 (Table 1). The *C. hyointestinalis* control strain was considered as non-*C. fetus* by both PCRs. It should be noted that this strain showed cross-reaction in PCR-2, i.e., amplification of the *ISC2* target (Table 1 and S2). For field isolates, 3 (4.7%) were identified as *Cff* and 61 (95.3%) as *Cfv* (Table 1). Isolates classified by PCR-1 and PCR-2 as *Cfv* were analysed by PCR-3 to identify them at biovar level. The control strain *Cfv* ATCC 25922 and *Cfvi3* were identified properly by PCR-3. For the field isolates, 52 (81.3%) were identified as *Cfvi* and 9 (14%) as *Cfv* “sensu stricto” (Table 1).

### Agreement between phenotypic and molecular identification tests

A moderate concordance (κ = 0.462; 0.256–0.669 CI) was observed between phenotypic and PCR identification tests for *C. fetus* subspecies identification (Table S3A). Discrepancies were found for 6 field isolates that were identified as *Cfv* by PCR but were classified phenotypically as *Cff* isolates (i.e., tolerance to 1% glycine and H_2_S production after 72 h and 5 days) (Table 1). Concerning the identification at biovar level, discordances were observed in the results obtained by the PCR-3 *versus* the H_2_S production at 72 h and 5 days. While at 72 h, 10 field isolates showed no H_2_S production, only 2 of these isolates (C1.43 and C3.5) remained as non-H_2_S producers at day 5 (Table 1). Therefore, the agreement between the PCR-3 and the H_2_S production test was moderate when H_2_S production was assessed after 72 h (κ = 0.553, 0.329–0.778 CI) and very good at day 5 (κ = 0.881, 0.631–1.000 CI) (Table S3B).

As described in the previous section, both PCR-1 and PCR-2 (Table 1) used for the identification of *Cff* and *Cfv* isolates showed 100% agreement (κ = 1.000; 0.755–1.000 CI) (Table S3C).

### *C. fetus* subspecies identification by WGS

The complete genome of the eight field isolates that showed discordant results in their identification by the phenotypic and PCR tests were sequenced. Taxonomic identification by KmerFinder showed a query coverage (the percentage of input query k-mers that match the template) of over 94.5% with strain CfviADRI545 (RefSeq: GCF_011601375.2) for all of them (Table 2). These taxonomic results were 100% concordant with the results obtained in the identification of these isolates by PCR-1 and PCR-2. The genes that code for the L-Cyst transporter (ATP-Bp, *yckJ* and *yckK*) were present in all isolates (100% coverage) and shared 100% identity in all cases except for the *yckK* gene in isolate C1.43 that had a mutation at position 2827 (Table 2). This mutation involved a Cytosine (C) to Thymine (T) transition, resulting in an amino acid change from serine to proline. However, this mutation has been observed in other H_2_S producing isolates (personal communication), suggesting that it does not involve significant changes in the functionality of the L-Cyst transporter. These results obtained from taxonomy analysis are therefore in perfect agreement with the amplification results obtained by PCR-3. In addition, the *cysK* gene, involved in the pathway of cysteine synthesis and H_2_S production, showed no mutations in the coding region or in its putative promoter region.

**Table 2:** Comparison of subspecies identification results using the different techniques (biochemistry, PCR, *WGS*, and gene sequencing) for discrepant *C. fetus* isolates

Sample	Phenotypic identification tests				PCR 1 & 2^a^	*WGS*^b^	PCR-3^c^	Gene sequence % identity^d^			
	**H**_**2**_**S** **72 h**	**H**_**2**_**S** **5 days**	**Glycine 1%**	**Phenotype**				**ATP_BP**	***yckJ***	***yckK***	***cysK***
**Sal3**	+	+	+	*Cff*	*Cfv*	*Cfv* (97.63)	*Cfvi*	100	100	100	100
**C3.46**	+	+	+	*Cff*	*Cfv*	*Cfv* (94.86)	*Cfvi*	100	100	100	100
**C3.52**	+	+	+	*Cff*	*Cfv*	*Cfv* (95.01)	*Cfvi*	100	100	100	100
**C2.6**	+	+	+	*Cff*	*Cfv*	Cfv (95.36)	*Cfvi*	100	100	100	100
**C3.4**	+	+	+	*Cff*	*Cfv*	*Cfv* (95.74)	*Cfvi*	100	100	100	100
**C3.10**	+	+	+	*Cff*	*Cfv*	*Cfv* (95.34)	*Cfvi*	100	100	100	100
**C1.43**	*-*	*-*	*-*	*Cfv*	*Cfv*	*Cfv* (95.89)	*Cfvi*	100	100	99.87	100
**C3.5**	*-*	*-*	*-*	*Cfv*	*Cfv*	*Cfv* (95.48)	*Cfvi*	100	100	100	100

*Cff Camylobacter fetus* subsp. *fetus*, *Cfv Camylobacter fetus* subsp. *venerealis*, *Cfvi Camylobacter fetus* subsp. *venerealis* bv*. intermedius*

^a^PCRs targeting the *nahE* and the *ISCfe* for *C. fetus* subspecies identification [14, 15]

^b^The number in brackets indicates the KamerFinder query coverage of these isolates compared to the reference strain *Cfvi*ADRI545

^c^PCR targeting the L-Cysteine transporter genes for *Cfv* biovars differentiation [16]

^d^Percentage identity result of these genes compared to the reference strain *Cff* 82–40

### Identification of *Campylobacter* in smegma field samples by MALDI-TOF and evaluation of the specificity of *C. fetus* molecular diagnostic methods

Seventy-five field isolates with morphology and motility resembling *Campylobacter* were obtained from smegma samples from 750 breeding bulls and identified by MALDI-TOF analysis as *Arcobacter butzleri* (*n* = 2), *Arcobacter cryaerophilus* (*n* = 4), *C. hyointestinalis* (*n* = 2), *C. sputorum* (*n* = 62) and *C. fetus* (*n* = 5). No cross-amplification reactions were observed for the non-*C. fetus* isolates when analysed by PCR-1 and PCR-2 (Table S4).

## Discussion

The accurate differentiation between the mammalian-associated *C. fetus* subspecies (*Cff* and *Cfv/Cfvi*) is crucial, since only *Cfv* and *Cfvi* cause BGC, a bovine sexually transmitted disease that produces infertility and great economic losses in the beef cattle sector [4, 5, 8, 17]. Several diagnostic methods are currently available to differentiate the *C. fetus* subspecies but none of them are 100% accurate due to the high homology between their genomes [2]*.* Biochemical tests (1% glycine tolerance and H_2_S production) [2, 18] and PCR amplification targeting the *C. fetus*-specific *nahE* gene and the *Cfv*-specific insertion element *ISCfe1* [14, 15] are the methods most commonly used for subspecies identification. However, several studies have revealed inconsistencies between phenotypic and molecular typing methods for the identification of *C. fetus* subspecies [19–22].

In this study, 64 Spanish *C. fetus* field isolates from breeding bulls were identified by phenotypic and PCR methods and the consistency of the results was compared. Considering the variability among isolates within each subspecies, only the results of the 1% glycine tolerance test, crucial for *C. fetus* subspecies differentiation (*Cff/Cfv*), and the H_2_S production test, that differentiates the biovars (*Cfv*/*Cfvi*), were considered for the final identification, as recommended [1, 2]. Here, a moderate concordance was observed between phenotypic and PCR for subspecies identification. The field isolates showing incongruent results were those phenotypically identified as *Cff* due to their tolerance to glycine and H_2_S production, despite carrying the *ISCfe1* insertion element characteristic of *Cfv*. This finding was also observed in the control strain *Cfvi3*, which is a glycine tolerant *Cfvi* strain. Previous studies have revealed the existence of *Cfv* isolates with the ability to grow on media supplemented with 1% glycine or higher, due to the acquisition of glycine tolerance related genes through phage mediated transduction or mutational processes [13, 19, 23]. In addition, glycine sensitive *Cff* isolates have also been described [23].

*Cfvi* is a phenotypic variant of *Cfv* with biochemical characteristics intermediate between *Cfv* and *Cff* [1]. H_2_S production allows differentiation between *Cfv “*sensu stricto*”* and *Cfvi*. *C. fetus*, and particularly *Cfv*, are slow-growing microorganisms that often need more than 48 h to develop well-formed colonies [1], therefore the WOAH recommends an incubation time of 72 h to assess H_2_S production [2]. In this study, we observed that the total number of H_2_S producing isolates increased by 13% when H_2_S production was measured after 5 days compared to 72 h, which could be related to its low metabolic activity. In fact, *C. fetus* has been described as a low or no H_2_S-producing species on standard media such as Triple Sugar Iron (TSI) and more sensitive and specific media are needed to detect its production [1, 24]. Therefore, the use of longer incubation periods under aseptic conditions seems to improve *Cfv* biovar identification. Actually, agreement between PCR-3 and H_2_S production was better when the latter was assessed at 5 days. However, after 5 days, two isolates still remained as H_2_S non-producers despite showing a *Cfvi* PCR pattern (i.e., 714 bp amplicon), suggesting the presence of a complete and functional L-Cyst transporter [16, 25].

In the present study, most *Cfv* isolates (81.3%) were identified as *Cfvi*, evidencing a predominance of this biotype in Spain, as described in South Africa and Argentina [26, 27]. A recent study, showed higher infectivity and long-term persistence of *Cfvi* in cattle compared to *Cff* and *Cfv*, suggesting better adaptation to the bovine genital tract or possible evasion of the immune response in cattle [28]. That study also reported differences in pregnancy rates in infected cows, being higher in *Cfvi* than in *Cfv*. Although genomic differences between *Cfv* and *Cfvi* have been identified [29], further studies are needed to determine which pathogenicity genes could be directly related to these differences in pregnancy rates.

Field isolates with inconsistent results were subjected to WGS. Several studies showed that WGS is an accurate method to correctly characterize *C. fetus* subspecies and to investigate their differences [5, 30, 31]. The taxonomic identification derived from genome analysis was in perfect agreement (100%) with the results obtained by PCR characterization of *C. fetus* subspecies. However, it is important to note that the *ISCfe1* insertion element used in PCR for *Cfv* identification can be transferred to other bacteria through bacterial conjugation, which could result in specificity issues [14, 32]. In addition, genome sequencing of the two H_2_S non-producing *Cfvi* isolates showed that they carried the complete L-cysteine transporter with no mutations in neither the genes nor the promoter region that could explain the non-production of H_2_S. To investigate the lack of H_2_S-production of these isolates, we analysed the *cysk* gene, closely related to the cysteine biosynthesis pathway in *C. jejuni* or the degradation of cysteine excess and H_2_S production in *Escherichia coli* [33, 34] that is also present in *C. fetus*. However, no mutations were found in this gene or its promoter region. Alternatively, the non-production in these *Cfvi* isolates could be partly related to the low levels of biochemical activity of *C. fetus* [2]. Nonetheless, further investigations regarding this biosynthetic pathway are needed to explain these results.

Here, 75 isolates obtained from the preputial area of breeding bulls were identified by MALDI-TOF as *A. butzleri*, *A. cryaerophilus*, *C. hyointestinalis*, *C. sputorum* and *C. fetus*. In cattle, these species may be part of the commensal microbiota. *Arcobacter* and *C. hyointestinalis* are mainly isolated from faecal samples of healthy cattle, although *Arcobacter* isolates have also been detected in preputial and bovine abortions, suggesting their implication in reproductive disorders in cattle [35–39]. The presence of these species in preputial samples might be due to faecal contamination [35]. On the other hand, *C. sputorum* is a commensal bacterium often found in genital tissues of healthy cattle and usually shares an ecological niche with *Cfv* [40]. In this study the most frequently isolated species was *C. sputorum* (82.6%), while only 6.6% of the isolates were identified as *C. fetus*. This low proportion of *C. fetus* could be explained because most of the bulls were sampled for routine control and had no reproductive problems. In addition, the physiological conditions and special nutritional requirements of *C. fetus* make it a poor competitor compared to other species, hampering its isolation [7, 41]. MALDI-TOF has gained importance in recent years in the diagnosis of microorganisms, due to its speed, accuracy and low-cost [42]. In this study, the application of the MALDI-TOF could only identify *C. fetus* isolates to the species level. Emele et al. [42] successfully differentiated between *C. fetus* isolates from reptiles (*C. fetus *subsp*. testudinum*) and mammals (*Cff*/*Cfv*) using MALDI-TOF, but differentiation between *Cff* and *Cfv* is not currently possible, suggesting that they also share a high similarity in their ribosomal protein spectra.

The transference of genetic material between bacteria inhabiting the same environment can be challenging for PCR-based identification methods. In fact, specificity problems due to cross-reactivity have been reported in most PCR methods available for *C. fetus* subspecies differentiation [14, 32]. Even the most efficient methods, such as those targeting the *nahE* gene and the *ISCfe1* insertion element, specificity problems have been reported associated to *C. hyointestinalis* isolates carrying the *ISCfe1* insertion element [14]. Here, none of the 70 field isolates tested showed cross-amplification reactions by PCR. However, only 2 *C. hyointestinalis* isolates were analysed. Thus, although these molecular identification methods seem to be reliable, it is important to emphasise the importance of combining targets specific for *C. fetus* (*nahE*) and *Cfv* (*ISCfe1*) to avoid misidentification, which at farm level could result in increased costs due to unnecessary treatment and culling of animals.

## Conclusions

This study confirmed the limitations of biochemical tests to correctly identify *C. fetus* subspecies and biovars. Yet, we showed that when prolonged incubation times (5 days) were used for H_2_S production tests, phenotypic identification was significantly improved. Instead, PCR-based methods (PCR-1 or PCR-2 for subspecies identification and PCR-3 for biovar identification) produced more accurate results. WGS and sequence analysis of the L-Cyst ABC Transporter and *cysK* genes, directly related to the H_2_S production, showed perfect agreement with PCR-based biovar identification results. WGS also confirmed the presence of the *ISCfe1* insertion element in all *Cf* subspecies *venerealis* isolates with discordant biochemical-PCR results thus confirming the PCR results for subspecies identification. However, cross-reactions can occur when the *ISCfe1* insertion element is acquired by other *Campylobacter* species, most commonly *C. hyointestinalis*. Nevertheless, other saprophytic microaerophilic bacteria that can be present in preputial samples did not cross-react. The identification of subspecies-specific genes would help to improve the diagnosis of BGC.

## Methods

### *Campylobacter fetus* isolates

A collection of 77 *C. fetus s*trains were used to compare phenotypic and molecular methods for both *C. fetus* subspecies and biovar identification. The collection included a reference strain from the American Type Culture Collection (ATCC) and a panel of 12 well-characterized strains obtained from the Faculty of Veterinary Medicine (Utrecht University, The Netherlands, National Reference Laboratory for Animal Campylobacteriosis) and NEIKER (Basque Country, Spain), that were used as positive controls and comprised: 1 *Cfv* and 1 *Cfvi* strains from bovine genital mucosa, and 6 and 5 *Cff* strains from bovine and ovine faecal origin, respectively. In addition, a *C. hyointestinalis* strain isolated from pig intestine was used as a negative control in the biochemical and molecular identification tests of *C. fetus* subspecies (Table 1). Another 64 field isolates, primarily obtained from preputial samples from breeding bulls subjected to BGC diagnosis in Spain, were obtained from the isolate collection of SALUVET laboratory (which provide services for BGC and bovine trichomonosis diagnosis to private veterinary practitioners in Spain) and the Spanish Reference Laboratory for Animal Campylobacteriosis: Laboratorio Central de Veterinaria (LCV)-Algete (MAPA: Ministry of Agriculture, Fisheries and Food, Madrid, Spain). LCV provides the abovementioned diagnostic services to official artificial insemination centres to comply with the animal health and traceability requirements for the authorisation of intra-Community movements of bovine-derived germ products. Metadata and previous identification of all isolates used are summarized in Table S5.

### Bacteriological culture and biochemical characterization of *C. fetus* isolates

All *C. fetus* isolates were subcultured on Columbia Agar plates supplemented with 5% sheep blood (Cos, Biomerieux, Marcy-l’Étoile, France) for 48 h at 37 ºC under microaerophilic conditions (5% O_2_, 10% CO_2_ y 85% N_2_, GENbox Microaer, Biomerieux, Marcy-l’Étoile, France) to obtain pure colonies. The phenotypical classification of the *C. fetus* isolates at the subspecies level was performed following standard protocols described by WOAH (Table S1) [2], as follows: growth at 42 °C, oxidase, catalase, H_2_S production in cysteine medium and tolerance to 3.5% sodium chloride (NaCl). The H_2_S production was measured both at 72 h and 5 days post inoculation. In order to avoid possible contamination that could alter the results, isolates were cultured under aseptic conditions in a hermetically closed anaerobiosis box, with an uninoculated vial as a negative growth control. Glycine tolerance was assessed on solid medium plates following the recommendations described elsewhere [43], with some modifications in medium composition: Nutrient Broth No. 2 (Oxoid, Hants, United Kingdom), 3% bacteriological agar (Bacto™ Agar, BD, Le Pont de Claix, France), 1% glycine (Glycine ≥ 99% molecular grade, Fisher Scientific, Waltham, MA USA) and 5% defibrinated sheep blood. For the growth test at 42 °C and tolerance to 1% glycine and 3.5% NaCl, all isolates showing a clear bacterial growth were considered positive. A colour changes from translucent to bluish was considered positive for the oxidase test. All isolates showing bubble formation on contact with H_2_O_2_ at 10 volumes were considered positive for catalase activity. Isolates that resulted in blackening of the lead acetate strips 5 days after inoculation were considered H_2_S producers.

Isolates were classified as *Cfv* when they showed negative results for both the 1% glycine tolerance and the H_2_S production tests; *Cff* when they showed 1% glycine tolerance independently of the H_2_S production; and *Cfvi* when they showed H_2_S production and glycine intolerance [1, 7, 19].

### DNA extraction and PCR assays

DNA extraction was performed from single colony cultures following the protocol for gram-negative bacteria of the DNeasy Blood and tissue kit (QIAGEN, Hilden, Germany). Identification of the isolates at subspecies level was carried out by a PCR (PCR-1) targeting the *C. fetus*-specific *nahE* gene and the *Cfv*-specific *ISCfe*1 insertion element using the following conditions: 1 × PCR buffer, 2.5 mM MgCl_2_, 0.1 mM of each dNTP, 0.6 µM of each primer and 1 U of Platinum™ Taq DNA Polymerase, 5 μl of DNA template, adjusted to 25 μl with nuclease-free water [15]. PCR-1 was performed in separate reactions for each target on a BioRad T100 thermal cycler using the cycling parameters described in the original publication by Abril et al. [15]. In addition, a multiplex real-time PCR (PCR-2) was performed targeting both the *nahE* gene and a different region (*ISC2*) of the *ISCfe*1 insertion element to rule out any misidentification resulting from the presence of mutations in the primer binding site of the *ISC1*, as described previously by van der Graaf et al. [14]. Each PCR reaction contained 10 μl of 2 × TaKaRa Ex Taq® DNA, 0.2 μl of ROX, 600 nM of each primer, 200 nM of each probe and 2 μl of DNA template, adjusted to 20 μl with nuclease-free water. The PCR-2 was carried out in a QuantStudio™ 5 Real-Time PCR instrument (Applied Biosystems), using the following cycling parameters: 95 °C for 30 s, followed by 40 cycles of 95 °C for 5 s and 60 °C for 34 s. Isolates were classified as *Cff* for each PCR when the *nahE* target was amplified in the absence of *ISCfe1* amplification. They were identified as *Cfv* when positive for both targets, and as non-*C. fetus* when they were negative for both targets. Moreover, isolates negative for *nahE* and positive for *ISCfe1* were considered as cross-reactions and therefore non-*C. fetus,* as previously described by Spence et al. [44].

Differentiation between *Cfv* and *Cfvi* isolates was performed by the PCR (PCR-3) described by Farace et al. [16] that targets the L-Cyst transporter containing ATP-Binding protein (ATP-Bp), permease protein (YckJ) and extracellular binding protein (YckK) coding genes. This transporter is directly related to H_2_S production and remains complete in *Cfvi* and *Cff* while *Cfv* strains show deletion of YckJ and partial deletion of YckK coding genes which makes them non-functional*.* The amplification mix consisted of 1 × PCR buffer, 1.5 mM MgCl2, 0.25 mM of each dNTP, 0.1 µM of each primer and 1.25 U of Platinum™ Taq DNA Polymerase, 5 μl of DNA template, adjusted to 25 μl with nuclease-free water. PCR-3 was performed on a BioRad T100 thermal cycler with the thermal cycler programme described in the original publication [16]. Isolates previously identified as *Cfv* by PCR-1 and PCR-2 were identified as *Cfvi* when they yielded a 714 bp amplicon in PCR-3 and as *Cfv “*sensu stricto*”* when the amplicon was 310 bp.

### Whole-Genome Sequencing and *C. fetus* subspecies identification

The whole genome of all *C. fetus* field isolates showing discordant results (*n* = 8) between biochemical and molecular identification was sequenced in a commercial facility (Eurofins genomics, Ebersberg, Germany). Libraries based on the NEBNext Ultra™ II FS DNA Library Prep Kit (Illumina) were prepared and sequenced using Illumina NovaSeq 6000 System to generate 2 × 150 bp paired-end reads. The preprocessing of the raw reads (quality check and filtering) was carried out as previously described [45]. Genome de novo assembly was performed using SPAdes v.3.15.3 [46] and the quality of the assemblies was assessed with QUAST v.5.0.2 [47], discarding contigs < 500 bp with PRINSEQ v.0.20.4 [48]. *C. fetus* subspecies taxonomic classification of the genomes was carried out by exact alignment of k-mers with KmerFinder v.3.0.2 [49–51] (database version 2022–07-11) by submitting the draft genome to the CGE website (https://cge.cbs.dtu.dk/services/KmerFinder, DTU Bioinformatics, Technical University of Denmark). Only reference strains identified by KmerFinder as predicted species with sequence coverage values above 80% were considered.

In addition, BLASTn v.2.10.1 + [52] and ABRicate v.1.0.1 (T. Seemann, https://github.com/tseemann/abricate) were used to screen for the sequences that encode for the three molecular components of the L-Cyst transporter (ATP-Bp, YckJ and YckK) and the cysteine synthase (CysK). For this, a custom nucleotide database was previously generated by extracting target regions from the reference strain Cff 82–40 (specifically ATP-Bp locus tags CFF8240_RS03845, *yckJ* CFF8240_RS03850, *yckK* CFF8240_RS03855, and *cysK* CFF8240_0922). When the sequence identity of the genes encoding the L-Cyst transporter or *cysK* was less than 100%, the sequences were aligned to the Cff 82–40 reference strain using Geneious Prime® (2023.0.3 v11.0.15 + 10).

### Isolation and identification of saprophytic microaerophilic bacteria in preputial samples from Spanish bulls

To further evaluate the specificity of the PCR-1 and PCR-2 assays for *C. fetus* subspecies identification, other saprophytic microaerophilic bacteria with morphology and motility resembling *Campylobacter* were isolated from smegma samples collected from 570 breeding bulls for routine STD diagnosis [10]. Briefly, the smegma samples were collected by preputial scraping of bulls in the field by veterinary practitioners, deposited in 5 ml tubes with *Campylobacter* transport medium, and sent to the SALUVET laboratory at room temperature within 24 h of collection. *Campylobacter* transport medium was formulated using thioglycolate broth (Sigma Aldrich) supplemented with *Campylobacter* growth supplement (Oxoid) and 7% foetal bovine serum (Thermo Fisher Scientific) without antibiotics. Upon arrival at the laboratory, samples were incubated at 36 °C for 24 h. After the incubation period, a sterile 0.65 µm filter was placed on Columbia Agar plates supplemented with 5% sheep blood (Cos, Biomerieux, Marcy-l'Etoile, France) and 200 µl of the upper portion of the C*ampylobacter* transport medium were inoculated into the centre of the filter. The filter was then removed, and the filtered sample was spread evenly over the entire surface of the agar plate using a sterile inoculation loop. The plates were then incubated for 48 h at 37 ºC under microaerophilic conditions (5% O_2_, 10% CO_2_ y 85% N_2_, GENbox Microaer, Biomerieux, Marcy-l’Étoile, France). Grown colonies were microscopically examined at 40 × magnification. Those showing morphology and motility resembling *Campylobacter* spp. were subcultured on Columbia Agar plates supplemented with 5% sheep blood (Cos, Biomerieux, Marcy-l'Etoile, France) and incubated for 48 h at 37 ºC under microaerophilic conditions. Subcultured isolates were then analysed by matrix assisted laser desorption ionization–time of flight (MALDI-TOF) mass spectrometry at the Microbial Identification and Characterization Unit at the Complutense University of Madrid (Spain) following the procedures detailed in Perez-Sancho et al. [53].

### Statistical analysis

Agreement between the results of the different assays for *C. fetus* subspecies and biovar differentiation was evaluated using the Cohen’s Kappa coefficient with a confidence level of 95%. To assess the strength of agreement between techniques the kappa values obtained were interpreted as follows: κ ≤ 0.20 = poor, 0.21–0.40 = fair, 0.41–0.60 = moderate, 0.61–0.80 = good and 0.81–1.00 = very good [54].

### Supplementary Information

**Additional file 1: Table S1.** Differential characteristics of *Campylobacter* spp. published in The Manual of Diagnostic Tests and Vaccines for Terrestrial Animals: Bovine genital campylobacteriosis; Chapter 3.4.4 [2]. **Table S2.** Phenotypic and molecular results of *C. fetus* isolates included in this study. **Table S3A.** Assessment of the concordance between phenotypic and molecular (PCR) tests for *C. fetus* subspecies identification. **Table S3B.** Assessment of the concordance between phenotypic and molecular (PCR) tests for *Cfv* biovars identification. **Table S3C.** Assessment of the concordance between molecular (PCR) tests for *C. fetus* subspecies identification. **Table S4.** Identification of isolates recovered from bull preputial smegma with morphology and motility resembling *Campylobacter* used in this study. **Table S5.** Metadata of all *C. fetus* isolates used in this study. **Table S6.** NCBI accession data of the eight *C. fetus* isolates sequenced in this study.

## Data Availability

The Whole Genome Sequencing project of *Campylobacter fetus* is deposited at GenBank under the BioProject accession number PRJNA1019261, which includes the 8 *Campylobacter fetus* genomes listed under the BioSample codes described in Table S6. Other datasets used and/or analysed during the current study are available from the corresponding author upon request.

## Abbreviations

*Cfv*: *Campylobacter fetus* subsp*. venerealis*
*Cfvi*: *Campylobacter fetus* subsp. *venerealis* bv. *intermedius*
*Cff*: *Campylobacter fetus* subsp. *fetus*
BGC: Bovine Genital Campylobacteriosis
STD: Sexual transmitted disease
WOAH: World Organisation for Animal Health
H_2_S: Hydrogen sulphide
L-Cyst: L-cysteine
WGS: Whole Genome Sequencing
NaCl: Sodium chloride
ATCC: American Type Culture Collection
LCV: Laboratorio Central de Veterinaria
